# Natural vertical cotransmission of Dengue virus and Chikungunya virus from *Aedes aegypti* in Brumado, Bahia, Brazil

**DOI:** 10.1590/0037-8682-0427-2021

**Published:** 2022-08-22

**Authors:** Henry Paul Granger, Cínthya Viana Souza Rocha, Thiago Macêdo Lopes Correia, Natalia Maria Pereira da Silva, Bárbara Aparecida Chaves, Nágila Francinete Costa Secundino, Paulo Filemon Paolucci Pimenta, Fabrício Freire de Melo

**Affiliations:** 1 Universidade Federal da Bahia, Instituto Multidisciplinar em Saúde, Vitória da Conquista, BA, Brasil.; 2 Fundação de Medicina Tropical Dr. Heitor Vieira Dourado, Diretoria de Ensino e Pesquisa, Manaus, AM, Brasil.; 3 Fundação de Medicina Tropical Dr. Heitor Vieira Dourado, Instituto de Pesquisas Clínicas Carlos Borborema, Manaus, AM, Brasil.; 4 Fundação Oswaldo Cruz, Instituto René Rachou, Belo Horizonte, MG, Brasil.

**Keywords:** Arboviruses, Coinfection, Infectious disease transmission, Vertical

## Abstract

**Background:**

Arthropod-borne viruses have recently emerged and are pathogens of various human diseases, including dengue, zika, and chikungunya viruses.

**Methods:**

We collected*Aedes aegypti*larvae (N = 20) from Brumado, Bahia, Brazil, and treated and individually preserved the specimens. We analyzed the samples for dengue, zika, and chikungunya viruses using molecular biology methods.

**Results:**

We found that 25% (N = 5) and 15% (N = 3) were positive exclusively for dengue and chikungunya viruses, respectively; 15% (N = 3) were coinfected with both.

**Conclusions:**

This is the first report of dengue and chikungunya virus coinfection in *A. aegypti* larvae.

In the past few decades, distinct arthropod-borne viruses (arboviruses) have emerged using arthropods as vectors and other animals as reservoirs. Some arboviruses are pathogens of various human diseases, including Dengue virus (DENV), Zika virus (ZIKV), and Chikungunya virus (CHIKV)^1^. Moreover, coinfection with these arboviruses in *Aedes aegypti* mosquitoes is possible^2^, including the ability to transmit more than one virus in a single bite^3^, highlighting their importance with regard to public health. Although horizontal transmission of arboviruses is well known, vertical transmission (VT)^4^ ^-^ ^6^ of these three arboviruses from *A. aegypti* have been reported, which may play an essential role in maintaining viruses in mosquitoes during interepidemic periods^6^.

Considering the importance of monitoring these arboviruses and their vectors, we evaluated *A. aegypti* larvae to identify natural modes of VT, coinfections, and which viruses were present among the different regions of Brumado city, which act as a predictor of potential epidemics and may be used by the health department.

Brumado is a town in the State of Bahia in Northeastern Brazil and the climate is semiarid, with an average annual temperature of 23.5 ^o^C and a rainy period from November to March^7^. According to the Brazilian Epidemiological Department, between November and December 40 dengue cases were reported; 28 were confirmed and 12 were discarded. Regarding zika, there were eight reported cases, six of which were confirmed and two were discarded. Regarding chikungunya, there were three reported and three discarded cases.

The city's Secretary of Health divides the area into three regions based on similar socioenvironmental and economic factors in accordance with the Brazilian Ministry of Health guideline. The larvae were individually and manually collected (stage L2 or L3) from these three regions in November and December 2019 by municipal health agents. After being washed three times in phosphate buffered saline, each larva where individually preserved in microtubes and sent to the Microbiology Laboratory of the Federal University of Bahia, Campus *Anísio Teixeira*. The larvae were identified using a dichotomous key^8^ and stored in a freezer at -70 ^o^C until processing.

No protected or privately owned land was accessed during larvae collection, and neither protected nor sensitive animals and plants were sampled. We obtained permits from the Brazilian *Instituto Chico Mendes de Conservação da Biodiversidade* and Ministry of Environment of Brazil (Registration number: 57,525) before collecting the mosquitoes.

RNA was extracted from macerated larvae using the commercial PureLink^®^ Viral RNA/DNA Mini Kit (Invitrogen, Thermo Fisher Scientific, Waltham, MA, USA) according to the manufacturer's guidelines. Using the High-Capacity RNA-to-cDNA^™^ Kit (Applied Biosystems, Thermo Fisher Scientific, Waltham, MA, USA), we synthesized the complementary (c)DNA until 2 μg of total viral RNA was obtained according to the manufacturer's instructions. A quantitative polymerase chain reaction assay was performed using Power Up^™^ SYBR^™^ Green Master Mix (Thermo Fisher Scientific, Waltham, MA, USA) and MicroAmp^™^ Optical 96-Well Reaction Plate (Applied Biosystems). Arbovirus-specific primer sets were designed for each arbovirus, as previously described (Table 1), and a standard curve was constructed using G-block amplification. The standard curve, samples, positive control (isolated cDNA from each arbovirus), and negative controls were allocated in duplicate to a 96-well plate. We adopted the following run parameters for all reactions: 40 cycles of fluorescence detection at 95 ^o^C for 15 s and annealing at 60 ^o^C for 1 min. Melting curves were generated for each run. All reactions were conducted in a StepOnePlus^™^ thermocycler (Applied Biosystems), and samples that showed specific amplification above the background threshold were considered positive. For positive samples, cycle threshold (Ct) values were compared with the respective standard curves to evaluate the number of viral cDNA replicates.

**TABLE 1:** Primer sets for each arbovirus.

Specificity	Name	Sequence (5' - 3')	Reference
Dengue virus	Forward	AGGACYAGAGGTTAGAGGAGA	13
	Reverse	CGYTCTGTGCCTGGAWTGAT	
Zika virus	Forward	CCGCTGCCCAACACAAG	14
	Reverse	CCACTAACGTTCTTTTGCAGACAT	
Chikungunya virus	Forward	TCGACGCGCCCTCTTTAA	15
	Reverse	ATCGAATGCACCGCACACT	

A total of 20 larvae (Figure 1A) were collected from the three administrative districts defined by the Health Department as follows: Region 1 (n = 10), Region 2 (n = 5), and Region 3 (n = 5). Of these, 55% (n = 11) tested positive for any of the three arboviruses, 25% (n = 5) were positive for DENV, and 15% (n = 3) were positive for CHIKV. In addition, 15% (n = 3) of the larvae were coinfected with DENV and CHIKV (Figure 1B). No samples were positive for ZIKV. Table 2 shows the Ct values and RNA copy numbers obtained from the positive samples.

**FIGURE 1:**
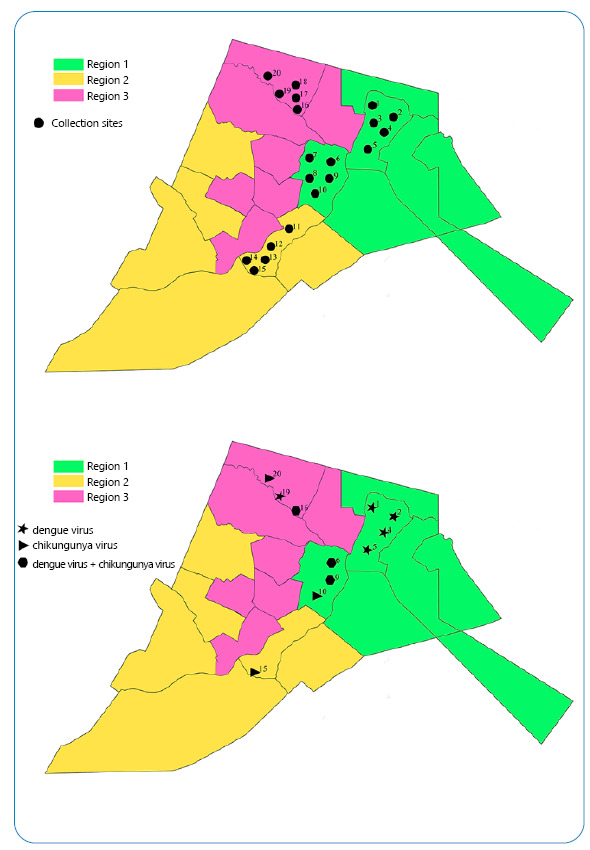
Larvae locations and arbovirus detection. Map of Brumado city in the Northeast region of Brazil **(A)** The city was divided into three regional administrative health units according to geographical areas. The black dots show the distribution of collection sites. **(B)** City map shows the collection points with positive larvae.

**TABLE 2:** Arboviruses detected in isolated larvae.

Larvae infection						
Positive larvae	Dengue virus cycle threshold	Copies/μl	Zika virus cycle threshold	Copies/μl	Chikungunya virus cycle threshold	Copies/μl
1	28.340	1,530.303	ND	ND	ND	ND
2	31.012	243.903	ND	ND	ND	ND
4	25.327	12,137.704	ND	ND	ND	ND
5	31.229	210.175	ND	ND	ND	ND
6	18.267	1,553,222.355	ND	ND	17.743	4,997,616.220
9	20.469	342,015.147	ND	ND	17.957	4,263,238.005
10	ND	ND	ND	ND	15.241	32,159,816.864
15	ND	ND	ND	ND	18.262	3,398,893.001
16	33.466	45.178	ND	ND	13.064	162,444,290.616
19	30.546	336.128	ND	ND	ND	ND
20	ND	ND	ND	ND	20.190	809,423.008

Our findings showed natural VT of these arboviruses in this vector during the rainy season in Brumado, which is consistent with previous studies on these larvae in Brazil^9^ ^,^ ^10^. Three coinfected samples were identified in Regions 1 and 3 with two distinct arboviruses, making this the first report of simultaneous coinfection of DENV and CHIKV in *A. aegypti* larvae.

Furthermore, in the coinfection cases, the number of viral copies of CHIKV was much higher than that of DENV. A study of mosquitoes in Mexico confirmed the coinfection capacity of DENV-2 (dengue virus serotype 2) and CHIKV and showed a similar disparity between the number of copies identified on the second and third day after exposure^11^. Between 5 and 15 days later, the presence of CHIKV stimulated DENV replication. Another study on *A. aegypti* mosquitoes in Mexico tested the possibility of coinfection among the three arboviruses. Exclusive DENV-2/CHIKV infection showed mild variation. Dissemination of DENV-2 was reduced 7 days after infection, and after only 14 days, a significant reduction (27%) in CHIKV transmission was observed^12^. A possible hypothesis based on nonstructural protein 1 has been proposed^2^, which is synthesized by the RNA of arboviruses from the genus Flavivirus, in our case DENV. This protein can reduce the immune response in mosquitoes, facilitating CHIKV replication during coinfection. 

Therefore, this study is the first to report the simultaneous coinfection of DENV and CHIKV in *A. aegypti* larvae. This finding, which is supported by our previous study^10^ in which we reported the coinfection of DENV and ZIKV in larvae from the same vector, demonstrates that the evaluation of larvae can be a crucial tool for controlling these arboviruses. Considering the high positivity rates observed in this study and detection of DENV and CHIKV, these diseases may have been underreported because the city only performs serological tests for dengue. Evaluating larvae is a quicker and more practical option than waiting for organisms, such as mosquitoes, to develop into adulthood. Recognizing which viruses are circulating among vector populations and their locations may help to predict epidemics by indicating probable emerging arboviruses, thereby directing public health actions.
